# Comparing the monetary value of a quality-adjusted life year from the payment card and the open-ended format

**DOI:** 10.1186/s12962-021-00298-0

**Published:** 2021-07-19

**Authors:** Ziping Ye, Fuyao Liu, Jia Ma, Ziyang Zhou, Chen Wang, Lihua Sun

**Affiliations:** College of business administration, Shenyang Pharmaceutical University, Shenyang, 110016 China

**Keywords:** Willingness-to-pay, Contingent valuation, Payment card, Open-ended question, Threshold

## Abstract

**Background:**

The payment card (PC) format and the open-ended (OE) format are common methods in eliciting willingness-to-pay (WTP) of one additional quality-adjusted life year (QALY). The aim of this research is to compare these two formats in eliciting the monetary value of a QALY.

**Methods:**

A contingent valuation survey was carried out using a pre-designed questionnaire with various hypothetical scenarios. The difference between the PC and the OE formats was evaluated by a two-sample equality test. Furthermore, generalized linear models were carried out to control observed heterogeneity and to test theoretical validity.

**Results:**

In total, 461 individuals were involved, among whom 235 (51%) answered the PC question, while 226 (49%) answered the OE question. Excluding zero response, the mean WTP values of these two formats for different scenarios varied dramatically, which was from 13,278 to 280,177 RMB for the PC, 18,119 to 620,913 RMB for the OE. The OE format tended to elicit lower values for less serious condition and higher values for more serious condition. However, equality test of mean and median demonstrated insignificant difference of these two formats for all scenarios. For both OE and PC format, most variables were found to have significant effect on the value of WTP/QALY. Moreover, joint estimation indicated a statistically significant positive effect on the OE results. Further analysis demonstrated that the imbalanced zero response distribution caused the main difference of these two formats.

**Conclusions:**

This research indicated insignificantly different WTP/QALY estimates of the PC format and OE format with the grouped data whereas significantly higher estimates of the OE format from the pooled data. These two formats were found to be valid. More research about the difference and the validity of various WTP eliciting methods would be recommended for a robust estimation of WTP/QALY.

**Supplementary Information:**

The online version contains supplementary material available at 10.1186/s12962-021-00298-0.

## Background

Budget allocation is one of the most prominent matters for decision makers in health-care systems today. The allocation is a complex, multifaceted issue. One of the vital questions, related to the budget allocation, is how much health-care systems should spend on the improvement of health-related outcome in terms of one additional quality-adjusted life year (QALY). Common decision rules of economic evaluations indicate that an intervention is believed to be cost-effective if the incremental cost-effectiveness ratio (ICER) of cost-effectiveness analysis (CEA) falls below the cost-effectiveness threshold value. Generally, there are two main approaches to this value, each taking a perspective from the demand side or supply side of the market [1, 2]. Those supply-side thresholds represent the health opportunity cost, indicating that whether a medicine is worthy of its cost depends on whether the amount of health-related outcomes it produces is larger than the health outcomes that could have been generated if some other medicine got funded [3]. Nevertheless, demand-side methods are in line with the method taken in other public sectors as well as a welfarist approach, where the monetary value of one additional QALY is estimated as willingness-to-pay per QALY (WTP/Q) by contingent valuation (CV) surveys. It is believed that WTP/Q can help improve efficiency in the margin within the healthcare sector as well as between sectors [4].

CV is usually used to elicit monetary values of a non-market good or service [5] by requesting participants to state their willingness-to-pay (WTP) for obtaining a good, in this context, for QALY (always a small amount). In the last decade, there are numerous studies estimating WTP/Q [6–13]. Typically, individuals have been asked about their WTP for health gains for which utility values were measured by EQ-5D population tariffs, Time-Trade-Off, Standard Gamble or Visual Analogue Scale.

However, great disparities exist in the type of health gain, respondents’ characteristics, and survey methodology—all of which may influence the perceived estimates of WTP/Q. Ryen et al. [14] included 24 studies and indicated that the WTP/Q value is significantly higher if the QALY gain comes from life extension rather than quality of life improvements. By comparing 2 similar surveys, Bobinac and colleagues [8] stated that WTP/Q is higher when the health gain in the survey scenario is uncertain. However, the impact of different CV questionnaire format has been barely investigated.

CV questionnaire format denotes the approach by which the respondent is required to provide their WTP, of which four classical techniques have been in use: iterative bidding, dichotomous choice, open-ended (OE) and payment card (PC) [15]. In this research, we focus on the latter two techniques, the OE and the PC format.

The PC technique was proposed by Mitchell [16] and first used in the general economics literature by Jones-Lee et al. [17]. Respondents were given a specific range of monetary values and asked to select the maximum value they would be willing to pay for a particular benefit. On account of the good performance of imitating real life by letting respondents ponder their WTP, the PC has become a prevalent method of eliciting WTP in health economics. The OE elicitation technique directly asks the respondent the maximum they would be willing to pay in a hypothetical scenario. As respondents are prone to anchoring on proposed values when the elicitation technique suggests the values, the OE method can lead to a more precise and independent WTP value than other elicitation techniques, as it does not suggest an answer [18]. It was further verified that the OE format is an effective technique if the final decision depends on a quantile instead of the mean [19].

There are several reasons why the PC and the OE method are chosen for this research. First of all, these two methods have been used broadly in estimating WTP per QALY [7–10]. Moreover, the advantages of using the PC and the OE method were that they were easier to understand and they required a short time for interviews, which is really important considering the respondent burden is a major concern due to the complexity of hypothetical scenario in estimating WTP/Q.

Given the popularity of the PC and the OE in health economics, more specifically, in estimating the monetary value of QALY, a plausible development is a direct comparison of these two formats. Although there is no research comparing these two methods in estimating WTP/QALY, studies have examined the discrepancies of eliciting methods in other fields. A general finding is that for health-related goods, the OE format causes lower WTP values [20, 21]. However, for environmental goods [22] or an ambulance helicopter service [23], relatively equal values were reported.

The aim of this research is straightforward, taking focus on the comparison of the PC and the OE formats. First, we examined the difference of WTP/QALY estimates from these two methods. Furthermore, we investigated the theoretical validity of each method to determine which method elicits more valid monetary value of QALY.

## Methods

### Study design and sample

We conducted a CV survey on general Chinese population between June 1st, 2019 and August 10th, 2019. A relatively low response rate was observed in the pilot study of the probability sample survey. Hence, quota sampling was used in the final survey with quotas based on sex, age, and income. First, study participants were recruited in-person by trained interviewers, then we interviewed those who satisfied the quotas. A questionnaire that measures maximum WTP per QALY for various hypothetical scenarios was used in this research. This survey was carried out with trained interviewers through telephone (a mobile app “WeChat”). Five different health statuses were defined using five-level EuroQol five-dimensional questionnaire (EQ-5D-5 L) descriptions [24, 25], including three treatment settings and two end-of-life scenarios. More details will be discussed in the next section. All subjects were asked for their full consent to participate in the study and no financial incentives were offered.

### Questionnaire

All together, we adopted 20 different questionnaires including 10 scenarios of each WTP technique. Each questionnaire contained 22 questions concerning quality of life, WTP, and demographic items as well as health-related issues. All questionnaires are identical except the part related to WTP. The demographic section included questions about age, sex, marital status, education, and family income. First, we evaluated the individuals’ present health state using the EQ-5D-5 L. Part 2 consisted of a hypothetical health state and a WTP exercise in which we asked individuals to state the maximum amount he or she would be willing to pay for treatment of a hypothetical condition. An example of part 2 can be found in the Additional file 1 [see Additional file 1]. To avert possible extreme WTP values and reveal general treatment in each scenario, small QALY gains, 0.2 QALY and 0.4 QALY, were applied in this research. Altogether, 10 eliciting scenarios were constructed (see Table 1).

**Table 1:** Scenarios of questionnaire

	Health state	No	EQ-5D-5 L description		QALY gain	Period (months)^a^
Treatment scenario	Mild	1	12,122	I have no problems in walking about; I have slight problems washing or dressing myself; I have no problems doing my usual activities; I have slight pain or discomfort; I am slightly anxious or depressed	0.2	15
	Mild	2			0.4	31
	Moderate	3	23,332	I have slight problems in walking about; I have moderate problems washing or dressing myself; I have moderate problems doing my usual activities; I have moderate pain or discomfort; I am slightly anxious or depressed	0.2	5
	Moderate	4			0.4	10
	Severe	5	44,332	I have severe problems in walking about; I have severe problems washing or dressing myself; I have moderate problems doing my usual activities; I have moderate pain or discomfort; I am slightly anxious or depressed	0.2	3
	Severe	6			0.4	6
Terminal illness		7			0.2	15
		8			0.4	26
Immediate death		9	11,115	I have no problems in walking about; I have no problems washing or dressing myself; I have no problems doing my usual activities; I have no pain or discomfort; I am extremely anxious or depressed	0.2	3
		10			0.4	6

^a^Since QALY = the period of life length (year) * utility of health state, the period was calculated as follows

For treatment scenarios, the period (month) = QALY gain/(utility of health state after treatment − utility of health state before treatment) * 12

Health state after treatment is perfect health, hence, the period (month) = QALY gain/ (1 − utility of health state before treatment) * 12

For terminal illness and immediate death, the treatment can prolong life expectancy in assumed health state, which should result in 0.2 or 0.4 QALY gain. Hence, for terminal illness the period (month) = QALY gain/utility of health state*12 + 3. For immediate death, QALY gain/utility of health state * 12

For treatment scenarios, a hypothetical scenario with description of EQ-5D-5 L (the health states mentioned in Table 1) was explained to participants. Without any treatment, they would live with the described health state for XX months. After XX months, they would fully recover. For each hypothetical health state, the WTP value was measured by the respondents’ willingness to purchase the treatment.

We also specified the following conditions to each respondent to clarify the assumed situation; (a) the treatment was not reimbursed by public health insurance, the full amount had to be paid beforehand; (b) loss of income due to the illness need not be considered (it is compensated by social security.); and (c) payment for the treatment will influence the respondents’ household.

“Terminal illness scenario” reflected the assumption that participants suffered a terminal disease with 3 months in severe health state (EQ-5D-5 L description: 44,332). A newly developed treatment could prolong life expectancy by 12 months (0.2 QALY) or 23 months (0.4 QALY) in that severe health state. For “immediate death scenario”, we assumed that because of fatal sickness, the respondents would die immediately. However, in this scenario we hypothesized that there was a treatment that could prolong life expectancy by 3 months (0.2 QALY) or 6 months (0.4 QALY) in health state 11,115.^1^

The WTP payment was defined as the amount of out-of-pocket expense to purchase an assumed intervention. Participants were asked if he or she would pay for the treatment. Those who replied “No” were then asked to give their reasons. If the answer was “yes”, the participant was requested to provide the maximum amount they were willing to pay out of pocket. The PC had the following categories: 3200 RMB (5% of Chinese GDP per capita, USD 457), 6450 RMB (10% of Chinese GDP per capita, USD 922), 12,900 RMB (20% of Chinese GDP per capita, USD 1,844), 25,800 RMB (40% of Chinese GDP per capita, USD 3,688), 51,600 RMB (80% of Chinese GDP per capita, USD 7,376), 77,400 RMB (120% of Chinese GDP per capita, USD 11,064), 103,200 RMB (160% of Chinese GDP per capita, USD 14,753). We sent the payment card to respondents before the survey started, and those who agreed to pay for the assumed intervention were asked to choose their maximum WTP from the payment card.

### Data analysis

Previous studies have applied two different methods of converting the data on WTP and QALY gains into WTP per QALY estimates, namely aggregated method and disaggregated method. The aggregated approach calculates the ratio by dividing the mean of WTP by the mean of QALY, whereas the disaggregated method estimates WTP/QALY for individuals, and subsequently estimates the mean value of WTP/QALY, which was proved to be a more appropriate method as it takes account of heterogeneity in preferences as well as individual’s marginal rate of substitution between health and money [26, 27]. Hence, the disaggregated method was applied in this research.

Descriptive statistics (mean, SD, median, inter-quartile range, minimum, maximum) for the WTP values of the PC and the OE formats were computed. Zero response of each format were compared and excluded for further analysis. We compared the mean and the median WTP/QALY obtained from the two elicitation methods of diverse scenarios using a two-sample equality test with bootstrapping.

Generalized linear models (GLMs) were carried out to control observed heterogeneity and test theoretical validity. In a broad sense, the theoretical validity of WTP/QALY estimates refers to whether the estimates concur with the underlying theory. The subsequent variables^2^ were selected for regression analysis in conformity with previous research [11–13]: age, income, hypothetical health state, and QALY gain. Age was proven to be a significant factor of WTP/QALY in previous research [11], indicating that being younger led to a higher WTP/QALY. Income is positively associated with WTP/QALY [12] and thus should be captured in the regression analysis. Furthermore, we also assumed that worse health state scenario [13] and smaller QALY gain should lead to a higher WTP/QALY [9]. For the base-case analysis, we included only positive WTP. For the further understanding of the difference of these two formats, we included all WTP responses, where zero WTP/QALY was converted into 1 RMB. In order to reduce the impact of outliers, the top 1% of values in both the OE and PC formats were trimmed for sensitive analysis. Moreover, we deleted all 18 samples which agreed to pay for intervention but did not give exact answers. Categorical variables were coded with dummy variables. We estimated GLMs with a log-link relationship. In order to choose an appropriate variance function for the GLMs, we performed modified Park test, which indicated gamma distribution. As for log link, this has the advantage of focusing on differences between groups of participants with respect to arithmetic rather than geometric means. Statistical analysis was performed with IBM SPSS version 23.0. and stata version 14.0.

## Results

### Respondent characteristics and summary statistics

Table 2 displays the demographic characteristics of respondents. In total, 461 individuals were involved, among whom 235 (51%) answered the PC question, while 226 (49%) answered the OE question. 61% of participants had a college degree. Around 35% of respondents had income less than 3000 RMB per month. Almost 19% participants in this research proclaimed that they were having some health problems. However, for all the dimensions in EQ-5D-5 L, most respondents reported no problem. The mean utility score of respondents was 0.95. A small portion of respondents (5%) had experienced hospitalization during the year. We found no significant differences between elicitation methods for all variables except education (p = 0.001).

**Table 2:** Respondents’ characteristics (N = 461)

Characteristic	Full sample	PC	OE
	Mean ± SD or N (%)	Mean ± SD or N (%)	Mean ± SD or N (%)
n	461 (100%)	235 (51%)	226 (49%)
Age	32.86 ± 11.84	31.92 ± 11.27	33.84 ± 12.36
Gender			
Male	219 (48%)	110 (47%)	109 (48%)
Female	242 (52%)	157 (53%)	117 (52%)
Education^a^			
≤ Primary school	29 (6%)	11 (5%)	18 (8%)
Secondary school	76 (17%)	25 (11%)	51 (23%)
High school	76 (17%)	39 (17%)	37 (16%)
≥ College	280 (61%)	160 (67%)	120 (53%)
Marital status			
Single	223 (48%)	105 (45%)	118 (52%)
Married	235 (51%)	130 (56%)	105 (47%)
Divorced/separated	2 (0%)	0 (0%)	2 (1%)
Widowed	1 (0%)	0 (0%)	1 (0%)
Income			
≤ 3000	162 (35%)	83 (35%)	79 (35%)
3001–5000	173 (38%)	85 (36%)	88 (39%)
> 5000	126 (27%)	67 (29%)	59 (26%)
Health utility	0.95 ± 0.07	0.95 ± 0.06	0.94 ± 0.09
Hospitalization experience during the year			
Yes	20 (5%)	7 (3%)	14 (6%)
No	441 (95%)	228 (97%)	213 (94%)
Health problems			
Yes	86 (19%)	44 (19%)	42 (19%)
No	375 (81%)	191 (81%)	184 (81%)

^a^Indicates there is significant difference between PC group and OE group on education

### Comparing formats with unconditional analysis

**Fig. 1:**
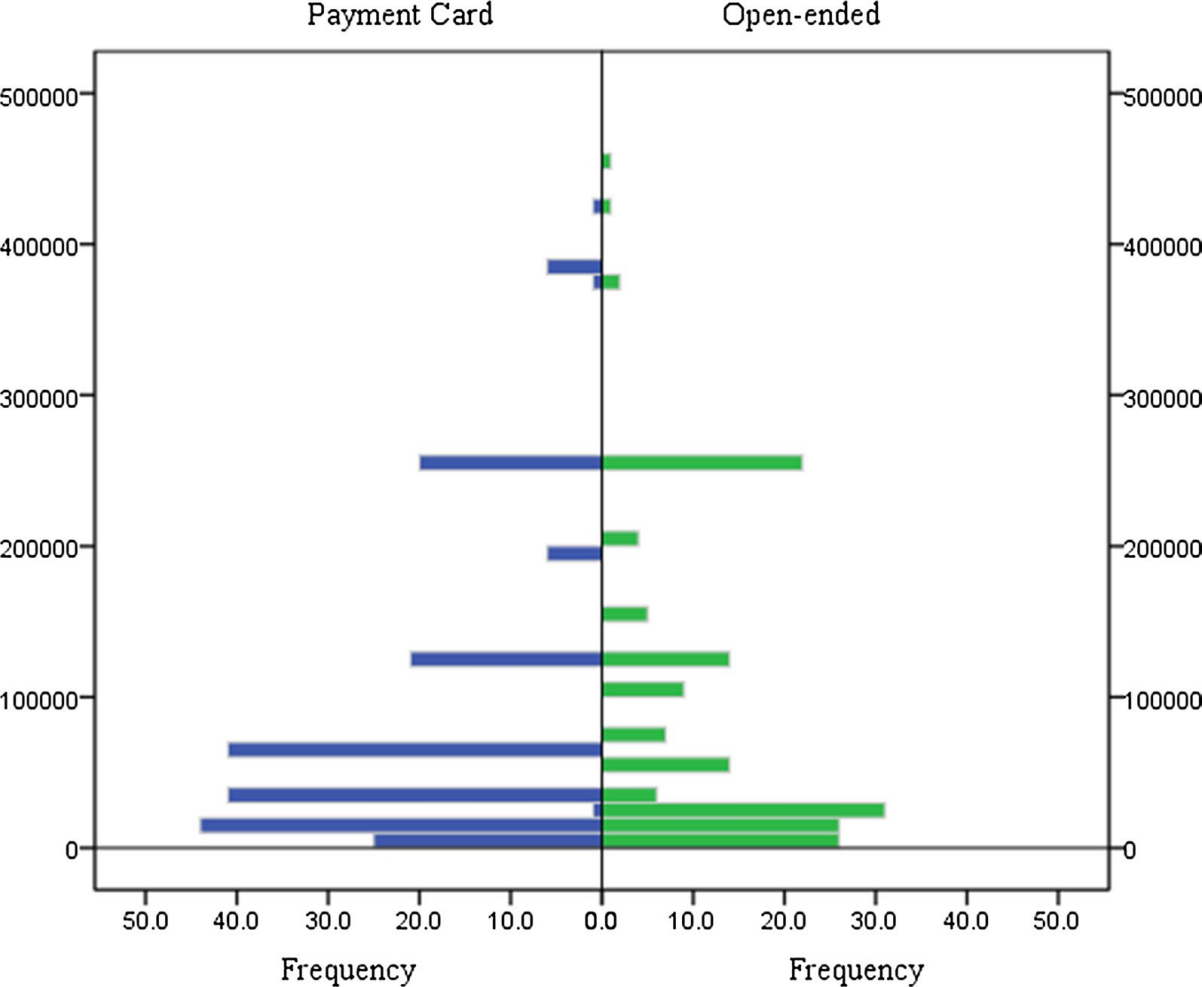
The distribution of willingness-to-pay per QALY of the PC and the OE formats

**Fig. 2:**
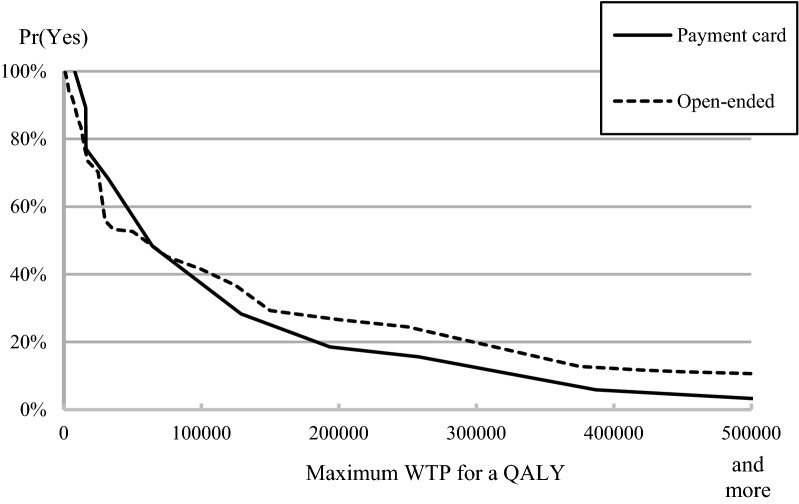
Proportion of accepted bids by elicitation format

The distribution of WTP/Q of the PC and the OE formats is displayed in Fig. 1. Furthermore, Table 3 presents descriptive statistics of WTP/QALY for the two elicitation methods. This research showed a small number of zero response, which is 12 (5.1%) for the PC, 34 (15.0%) for the OE. Detailed information about zero response can be found at Table 3. The range of median values for PC format for different questionnaires is 8000 to 258,000 RMB, whereas for OE is 7500 to 500,000 RMB. Figure 2 displays the ratio of accepted bids according to the elicitation method. These two crossing lines indicated that the OE format tended to elicit more extreme values, though the difference between two elicitation methods did not seem to be substantial.

**Table 3:** Descriptive statistics and equality tests of WTP per QALY using PC and OE formats

Questionnaires	WTP technique	N	Zero response, N (%)	Mean	Standard deviation	Median	Minimum	Maximum
Mild, 0.2 QALY	PC	26	1 (3.8%)	37,890	32,658	28,426	500	129,000
	OE	25	4 (16%)	25,476	20,243	23,000	5000	100,000
Mild, 0.4 QALY	PC	25	0 (0%)	21,595	12,914	16,125	8000	64,500
	OE	25	4 (16%)	18,119	27,211	7500	500	125,000
Moderate, 0.2 QALY	PC	21	0 (0%)	75,631	107,888	64,500	16,000	516,000
	OE	21	2 (10%)	105,447	125,083	100,000	2500	516,000
Moderate, 0.4 QALY	PC	23	1 (4.3%)	51,295	54,553	32,250	8000	258,000
	OE	22	3 (13.6%)	84,934	83,739	75,000	1250	250,000
Severe, 0.2 QALY	PC	22	0 (0%)	34,409	18,187	32,250	16,000	64,500
	OE	19	2 (11%)	40,000	36,827	25,000	10,000	150,000
Severe, 0.4 QALY	PC	23	5 (21.7%)	13,278	9666	8000	750	32,250
	OE	20	1 (5%)	19,868	20,331	12,500	1250	75,000
Terminal illness, 0.2 QALY	PC	25	1 (4%)	280,177	219,279	258,000	16,000	1,000,000
	OE	18	11 (61%)	570,714	413,727	420,000	75,000	1,000,000
Terminal illness, 0.4 QALY	PC	26	1 (4%)	232,600	120,808	258,000	64,500	516,000
	OE	25	0 (0%)	221,000	127,614	200,000	25,000	500,000
Immediate death, 0.2 QALY	PC	19	2 (11%)	273,647	237,721	129,000	16,000	750,000
	OE	26	3 (12%)	620,913	1,019,954	500,000	15,000	5,000,000
Immediate death, 0.4 QALY	PC	25	1 (4%)	189,984	286,863	64,500	8000	1,250,000
	OE	25	4 (16%)	242,381	235,387	250,000	7500	750,000

*PC* payment card, *OE* open-ended

The results of equality tests of mean and median were presented in Table 4, which were, to some degree, consistent with the figure of the ratio of accepted bids. The general tendency was that for mild health state scenario, the PC yielded higher mean value, whereas for all other four health scenarios, the OE method was witnessed with much bigger mean WTP/QALY valued except terminal illness scenario with 0.4 QALY gain. Equality test of mean with bootstrap indicated insignificant difference of the means of these two techniques for all types of questionnaires. No differences were found in the median for these two formats in all five scenarios

**Table 4:** Comparison between the PC and the OE formats by different scenarios

Questionnaires	Mean Difference	95% Interval of mean difference by bootstrap	Equality test of mean (bootstrap)	Equality test of median
Mild, 0.2 QALY	12,413	(− 2621, 27,294)	0.141	0.067
Mild, 0.4 QALY	3476	(− 10,464, 14,469)	0.598	0.903
Moderate, 0.2 QALY	− 29,816	(− 108,607, 41,299)	0.477	0.595
Moderate, 0.4 QALY	− 33,639	(− 82,653, 9596)	0.165	0.662
Severe, 0.2 QALY	− 5590	(− 25,971, 12,205)	0.596	0.917
Severe, 0.4 QALY	− 6591	(− 17,302, 2750)	0.22	0.408
Terminal illness, 0.2 QALY	− 290,537	(− 638,371, 28,961)	0.113	0.623
Terminal illness, 0.4 QALY	11,600	(− 58,726, 78,265)	0.728	0.777
Immediate death, 0.2 QALY	− 347,266	(− 865,403, − 3927)	0.28	0.522
Immediate death, 0.4 QALY	− 52,396	(− 200,299, 104,131)	0.51	0.309

### Comparing formats with conditional analysis

#### Separate estimations by elicitation format

For each elicitation method we looked for the determinants of WTP/QALY with GLMs (Table 5). For both OE and PC format, most variables were found to have significant effect on the value of WTP/QALY. The monetary value of QALY was proved to be significantly influenced by valuation scenarios in both models, while participants were prepared to pay more for more serious conditions, even though the difference of WTP/QALY values for terminal illness and base-case group (immediate death) was not significant in both formats. Furthermore, we confirmed that for both formats, smaller QALY gain led to a higher WTP/QALY. We found a positive effect of income on WTP/QALY for both formats—which argues for the validity of the stated-preference survey [24]. Age was assumed to be negatively related to participants’ WTP: as respondents’ age increase, their WTP/QALY decreased. However, for the PC technique, participants’ age was not a statistically significant variable, whereas for the OE format, age was a statistically significant factor. The sensitivity analysis proved the robustness of these findings, which can be found in the Additional file 2.

#### Joint estimation over the two elicitation formats

We studied the impact of the elicitation technique on WTP over the whole sample by introducing dummy variables for the OE format (the PC format as the reference). The GLM with whole sample including only positive WTP indicated statistically significant positive effect on the OE results, demonstrating that OE format has higher chance to elicit higher WTP/QALY than the PC format. Nevertheless, when we converted all zero WTP/Q into 1 RMB, the difference between these two formats became insignificant. We may conclude that the imbalanced zero response distribution cause the main difference of these two formats. Regarding the determinants of WTP, the joint estimation confirms previous results: a significant and negative effect of age and QALY gain, a significant and positive effect of income. WTP/QALY was also proved to be affected by valuation scenarios.

**Table 5:** Generalized linear models for WTP/QALY values

	All, n = 415			ALL, including zero WTP			PC, n = 223			OE, n = 192		
	Coef.	Std. Err.	P > z	Coef.	Std. Err.	P > z	Coef.	Std. Err.	P > z	Coef.	Std. Err.	P > z
OE (vs. PC)	0.21	0.10	0.03*	0.09	0.1	0.38						
Scenario (vs. Immediate death)												
Mild	− 2.44	0.15	0.00**	− 2.45	0.16	0.00**	− 2.16	0.19	0.00**	− 2.80	0.22	0.00**
Moderate	− 1.39	0.15	0.00**	− 1.35	0.16	0.00**	− 1.52	0.20	0.00**	− 1.24	0.23	0.00**
Severe	− 2.35	0.15	0.00**	− 2.33	0.16	0.00**	− 2.27	0.20	0.00**	− 2.42	0.23	0.00**
Terminal illness	0.00	0.15	0.99	− 0.07	0.16	0.66	0.06	0.19	0.76	− 0.10	0.23	0.66
QALY gain (vs. 0.2 QALY)												
0.4 QALY	− 0.61	0.10	0.00**	− 0.57	0.1	0.00**	− 0.48	0.12	0.00**	− 0.72	0.15	0.00**
Income (vs. ≤ 3000)												
3001–5000	0.21	0.11	0.06	0.23	0.12	0.06	0.16	0.14	0.27	0.21	0.18	0.24
> 5000	0.68	0.12	0.00**	0.69	0.13	0.00**	0.61	0.16	0.00**	0.76	0.20	0.00**
Age (vs. < 25)												
25–39	− 0.14	0.12	0.24	− 0.11	0.12	0.38	0.10	0.15	0.51	− 0.42	0.18	0.02*
≥ 40	− 0.36	0.12	0.00**	− 0.39	0.12	0.00**	− 0.25	0.15	0.11	− 0.49	0.18	0.01*
Constant	12.63	0.16	0.00**	12.55	0.17	0.00**	12.41	0.18	0.00**	13.05	0.25	0.00**

*PC* payment card, *OE* open-ended, *QALY* quality-adjusted life year

^*^ Indicates that there are statistically significant differences at the 5% level; ^**^ indicates that there are statistically significant differences at the 1% level

## Discussion

We compared WTP/QALY estimates generated from the PC format and the OE format and found that the mean WTP values of these two formats varied dramatically for different scenarios and QALY gains. The OE format tended to elicit more extreme values, indicating that for mild health state scenario, the PC yielded higher mean value, whereas for all other four health scenarios, the OE method was witnessed with much bigger mean WTP/QALY. However, equality test of mean and median demonstrated insignificant difference of these two formats for all scenarios. GLM regression demonstrated the validity of both and statistically significant positive effect on the OE results. Further analysis indicated that the imbalanced zero response distribution caused the main difference of these two formats.

The theoretical validity can be examined by determining whether the results are consistent with theoretical constructs. Probing the theoretical validity is the most popular test of validity applied to stated-preference techniques mostly since it is comparatively easy to perform. The performance of both PC and OE format appears to be highly satisfactory, whereas the PC format failed to comply with the assumption that being younger leads to a higher WTP/QALY. In comparison with the PC format, the OE technique had a stronger association with most variables in the regression model. In theory, the PC question tends to cause range bias. In the OE form, only after a careful reflection can respondents answer WTP question [18], which might be a fundamental procedure in assessing the value of health.

We did not pool all the data and present overall mean WTP/QALY estimates for each format because of the variation of mean WTP/QALY for different scenarios with different QALY gain. Instead, detailed information of descriptive statistics of different types of questionnaires were reported for each format. It was found that the OE format was related with lower WTP/QALY for less serious condition and higher values for more serious condition. Moreover, the results of GLM regressions demonstrated that the OE format tended to higher WTP/QALY, which is inconsistent from previous research in healthcare. By asking questions of women’s WTP for a screen process, Donaldson et al. [20] concluded that the PC format was related with higher mean and median WTP, which was proved again by the study on colorectal cancer screening of Whynes and colleagues [21]. We may argue here that body screening as well as mild treatment scenario in this research could be considered as less serious condition, where the OE format tended to elicit lower values.

This is the first study to compare WTP/QALY estimates generated from the PC and the OE formats. However, we have encountered certain practical limitations. First, only the theoretical validity of the two eliciting methods was performed; essential elements like external validity and reliability were not assessed in this study. Second, quota sampling instead of probability sampling was applied in this research. Hence, the participants used in this study may not be a perfect representation of the Chinese population. Due to the cognitive challenge of this type of survey, most studies of WTP include a sample with a higher education compared to the general population [28, 29]. In this study, those with higher levels of education were over-represented. However, we found that education level had no significant impact on WTP/QALY. Hence, the potential bias in the WTP estimate is likely to be minor. Third, according to previous research [4], respondents’ health state, especially those who had health state worse than the hypothetical questions, might influence their WTP for a QALY, but we did not perform any analysis regarding to this issue since most participants stated perfect health. There were 5 participants whose health state is worse than the illness state in the hypothetical questions, all came from mild treatment scenario (two for PC group and three for OE group). One refused to pay anything for the treatment, the other four state plausible payment. Moreover, we did not distinguish genuine zeros from protest responses, which might potentially bias our conclusion. Finally, a critical limitation is the imaginary nature of all WTP surveys [30]. Like other studies, the participants might find it hard to picture a hypothetical health state that significantly differs from conditions that they have experienced before. More research about the validity of various WTP eliciting methods would be needed for a robust estimation of monetary value per QALY.

## Conclusions

The study compared WTP/QALY estimates generated from the PC and the OE formats and found that the OE format tended to elicit lower values for less serious condition and higher values for more serious condition. However, equality test of mean and median demonstrated insignificant difference of these two formats for all scenarios. GLM regression demonstrated the validity of both and statistically significant positive effect on the OE results. More research about the validity of various WTP eliciting methods would be needed for a robust estimation of WTP/QALY.

## Supplementary Information

**Additional file 1.** An example of willing-to-pay question. This is an example of part 2 of the questionnaire, which contains a hypothetical health state and a WTP exercise. Individuals was asked to state the maximum amount he or she would be willing to pay for treatment for a hypothetical condition.

**Additional file 2.** Sensitive analysis of generalized linear models for WTP/QALY values. This is the result of sensitive analysis of GLMs.

## Data Availability

The datasets used during the current study are available from the corresponding author on reasonable request.

## Abbreviations

PC: Payment card
OE: Open-ended
WTP: Willingness-to-pay
QALY: Quality-adjusted life year
ICER: Incremental cost-effectiveness ratio
CEA: Cost-effectiveness analysis
CV: Contingent valuation
EQ-5D-5L: Five-level EuroQol five-dimensional questionnaire

## Footnotes

1 We used perfect health (11,111) in the pilot study, which was believed to be too ideal since most people feel extreme anxious in face of death. Hence, 11,115 (5 means extreme anxious or depressed) was used for the immediate death scenario.

2 The regressors were chosen based on the bivariate analysis (the Mann-Whitney U-tests for dichotomous variables, Kruskal-Wallis H tests for polychromous variables, Spearman’s rank correlation coefficient for the continuous variable) as well as previous researches.
