# Supplementary material for: Comparing the monetary value of a quality-adjusted life year from the payment card and the open-ended format
Source: Cost Eff Resour Alloc. 2021 Jul 19;19:45. doi: 10.1186/s12962-021-00298-0 (PMC8287741; doi:10.1186/s12962-021-00298-0)
Supplement: Supplementary file 1 — Additional file 1. An example of willing-to-pay question. This is an example of part 2 of the questionnaire, which contains a hypothetical health state and a WTP exercise. Individuals was asked to state the maximum amount he or she would be willing to pay for treatment for a hypothetical condition. [file 12962_2021_298_MOESM1_ESM.docx]

Please imagine that you are having following symptoms: severe problems in walking about, severe problems washing or dressing yourself, moderate problems doing your usual activities, moderate pain or discomfort and slightly anxious or depressed (symptom image should be sent at the same time and remind the interviewee of this image), If you do not receive any treatment, you will recover perfect health status after you live for 3 months in this health status.

SYMPTOM IMAG

| I have **severe** problems in walking about | 3 Months | I have **no** problems in walking about |
| --- | --- | --- |
| I have **severe** problems washing or dressing myself |  | I have **no** problems washing or dressing myself |
| I have **moderate** problems doing my usual activities |  | I have **no** problems doing my usual activities |
| I have **moderate** pain or discomfort |  | I have **no** pain or discomfort |
| I am **slightly** anxious or depressed |  | I am **not** anxious or depressed |

Now a new treatment has been developed. You can immediately recover perfect health status if you receive the new treatment. However, this treatment is not reimbursed by public health insurance. You will have pay full amount out of your pocket to receive the treatment. We assume that your income remains the same.

Q1: Would you purchase the treatment? [Yes/No]

Q2 for the PC: (Send the image of payment card first)

How much maximum will you pay for this new treatment according to the image I just sent you? (read these numbers aloud only under request)

PAYMENT CARD IMAGE

□3200 □6450 □1 2900 □2 5800 □5 1600 □7 7400 □10 3200

Q2 for the OE:

How much maximum will you pay for this new treatment?
